# NHS health checks through general practice: randomised trial of population cardiovascular risk reduction

**DOI:** 10.1186/1471-2458-12-944

**Published:** 2012-11-01

**Authors:** Thomas Cochrane, Rachel Davey, Zafar Iqbal, Christopher Gidlow, Jagdish Kumar, Ruth Chambers, Yvonne Mawby

**Affiliations:** 1 Centre for Research and Action in Public Health, Faculty of Health, University of Canberra, Canberra ACT2601, Australia; 2 NHS Stoke on Trent, Directorate Public Health, Civic Centre, Glebe Street, Stoke on Trent, ST4 1HH, United Kingdom; 3 Centre for Sport, Health and Exercise Research, Staffordshire University, Leek Road Campus, Stoke on Trent, ST4 2DF, United Kingdom

**Keywords:** Vascular disease, Lifestyle change, Prevention, Multi-factorial risk, Policy implementation

## Abstract

**Background:**

The global burden of the major vascular diseases is projected to rise and to remain the dominant non-communicable disease cluster well into the twenty first century. The Department of Health in England has developed the NHS Health Check service as a policy initiative to reduce population vascular disease risk. The aims of this study were to monitor population changes in cardiovascular disease (CVD) risk factors over the first year of the new service and to assess the value of tailored lifestyle support, including motivational interview with ongoing support and referral to other services.

**Methods:**

Randomised trial comparing NHS Health Check service only with NHS Health Check service plus additional lifestyle support in Stoke on Trent, England. Thirty eight general practices and 601 (365 usual care, 236 additional lifestyle support) patients were recruited and randomised independently between September 2009 and February 2010. Changes in population CVD risk between baseline and one year follow-up were compared, using intention-to-treat analysis. The primary outcome was the Framingham 10 year CVD risk score. Secondary outcomes included individual modifiable risk measures and prevalence of individual risk categories. Additional lifestyle support included referral to a lifestyle coach and free sessions as needed for: weight management, physical activity, cook and eat and positive thinking.

**Results:**

Average population CVD risk decreased from 32.9% to 29.4% (p <0.001) in the NHS Health Check only group and from 31.9% to 29.2% (p <0.001) in the NHS Health Check plus additional lifestyle support group. There was no significant difference between the two groups at either measurement point. Prevalence of high blood pressure, high cholesterol and smoking were reduced significantly (p <0.01) in both groups. Prevalence of central obesity was reduced significantly (p <0.01) in the group receiving additional lifestyle support but not in the NHS Health Check only group.

**Conclusions:**

The NHS Health Check service in Stoke on Trent resulted in significant reduction in estimated population CVD risk. There was no evidence of further benefit of the additional lifestyle support services in terms of absolute CVD risk reduction.

## Background

Despite many advances in treatment, the global burden of the vascular diseases (including heart disease, stroke and other disorders with a strong vascular component, such as diabetes and chronic kidney disease) is projected to rise and to remain the dominant non-communicable disease cluster well into the 21^st^ century [1-3]. This challenge to global health has prompted calls for renewed efforts to tackle the lifestyle and treatment inertia issues that are known to be impediments to successful prevention of these diseases [4]. Over the past 40 years since the global pandemic of atherosclerotic diseases became widely accepted [5], research support for the value of prevention has been greatly strengthened but this evidence has yet to translate to successful public health policies that demonstrate a down-turn in the population burden of these diseases. Evidence alone is insufficient to effect change.

More recently, it has become clear that effective public health policy for the prevention of the major vascular diseases will require concerted partnership and action among multiple agencies, both within and outside the traditional health sector [6,7]. In a shift to a more concerted, multi-sector policy for prevention, the Department of Health in England introduced the NHS Health Check programme in 2009 [8]. Since this programme is in its infancy there is a need for reliable evidence on recruitment to the programme and outcomes following implementation of the guidelines.

There is abundant evidence that lifestyle change can have a beneficial effect on individual vascular disease risk factors. For example, high blood pressure can be prevented with dietary intervention alone [9], diabetes can be prevented or delayed with diet and physical activity in combination [10] and population levels of cholesterol can be reduced with diet change alone or in combination with medication [11]. However, it has been argued that, because of the cumulative effect of multiple risk factors, vascular risk would be best managed using multi-factorial estimates of absolute risk based on established risk estimators such as the Framingham equations for estimating cardiovascular risk [12,13]. Indeed, there is research evidence to support the benefits of such an approach [14-16] but routine use in clinical practice has yet to be established. Further evidence is required to convince patients and health professionals that greater benefits can be achieved by adopting a multi-factorial approach, coupling medications (e.g. for blood pressure and/or cholesterol control) with more concerted efforts to change established risk behaviours related to lifestyle, such as tobacco use, unhealthy diet, insufficient physical activity and excessive use of alcohol.

As far as we are aware, the NHS Health Check initiative in England is the first national programme to attempt to develop a systematic and coordinated approach to the management of major vascular disease risk at the population level. The initiative is led in most areas by general practices, which are the custodians of clinical data and the first points of call with regard to health issues for most people in England. This paper evaluates the change in population cardiovascular disease (CVD) risk following implementation of NHS Health Check in Stoke on Trent after one year of the programme. The question of whether there is added value of lifestyle support over and above the basic health check in reducing population CVD risk is also addressed.

## Methods

### Study design

A randomised trial design was used comparing patients who received the NHS Health Check only service with patients who received an NHS Health Check plus additional support for lifestyle change. Full details of the methods have been published elsewhere [17]. Modifications to the original protocol and a summary of the details of methods pertinent to the research reported here are summarised below. All participants gave written informed consent and the study was approved by the South Birmingham Research Ethics Committee, West Midlands Region of England.

### Settings and participants

Thirty eight general practices were recruited to the study between September 2009 and February 2010. A total of 601 participants were recruited to the trial, 365 to the NHS Health Check only group and 236 to the NHS Health Check plus additional lifestyle support group. Dedicated software, Oberoi Clinical Observations (Oberoi Consulting, Derby, UK), was used to stratify practice lists by estimated 10-year CVD risk. Patients with an estimated CVD risk of ≥ 20% were considered eligible for inclusion in the trial. Each practice list was randomised into trial groups by an independent researcher, blinded to any further details of patients or practices. Practice nurses or project support workers in each practice went through their list, systematically contacting patients in batches of 20–50 depending on practice size, until either the recruitment target for the practice had been reached or all eligible patients had been invited. Up to three reminder letters were sent before a non-response was recorded. Allocation to group was only revealed once all details of the trial had been explained to the patient and written informed consent to participate had been given at the end of the final NHS Health Check assessment visit.

### Interventions compared

The NHS Health Check group received an NHS Health Check and usual general practice care, including medication and referral to smoking cessation services, dependent upon the outcome of the health check but did not receive additional lifestyle support. The NHS Health Check plus additional lifestyle support group received an NHS Health Check and were also offered additional support for lifestyle change. This support, which was based on the national Health Trainer motivational interview/counselling model, included: one to one consultation with a lifestyle coach, the opportunity to discuss, develop and negotiate a personalised health improvement plan and lifestyle improvement priorities identified by the patient and referral to free support sessions for: weight management, physical activity, cook and eat and positive thinking, as desired by the individual participant. Additional support (including up to six hours of one to one contact if needed) was provided for 20 weeks in the first instance with ongoing support available for up to a year if required. Full details of the primary care interventions are given in the Primary Care Toolkit [18].

### Outcome measures

The primary outcome measure for the evaluation was the Framingham 10-year CVD risk using the Joint British Societies’ Guidelines [19], which was the recommended procedure at the time of conducting this research. Secondary outcome measures included changes in individual CVD risk factor categories (high blood pressure, high cholesterol and smoking) as well as changes in lifestyle related risk factors of weight, body mass index (BMI), diet and physical activity. Blood pressure, height, weight and waist circumference were measured in standardised fashion by trained nurses. Total cholesterol, high density lipoprotein (HDL) and fasting or random plasma glucose (for confirmation of diabetes status, the nature of the test used depending on practice preference) levels were measured as per protocol. For the purposes of this evaluation, high blood pressure was defined as having a systolic blood pressure ≥ 140 and a diastolic blood pressure ≥ 90 mmHg, high cholesterol was defined as having total cholesterol to HDL ratio ≥ 4.5, overweight was defined as having BMI ≥ 25 and < 30 kgm^-2^ and obesity was defined as having BMI ≥ 30 kgm^-2^. Central obesity was defined from waist circumference measurements as follows: males – waist circumference ≥ 102 cm (Asian males ≥ 90 cm), females ≥ 88 cm (Asian females ≥ 80 cm). Smokers were defined as those who were current smokers or who had given up in the last year. Finally, diet was categorised for each patient into 1 – poor, 2 - average or 3 - good using guideline diets outlined in the Primary Prevention Toolkit (Appendix 1c) and habitual physical activity was categorised into one of 4 groups, 1 - inactive, 2 - moderately inactive, 3 - moderately active and 4 -active, using the General Practice Physical Activity Questionnaire [20]. It is acknowledged that both the diet grade and exercise grade measures are relatively crude and lack the sensitivity to detect subtle changes in either diet or physical activity that may benefit health. On the other hand, they are simple to use measures that capture the importance of lifestyle to cardiovascular health and serve as a focus for discussion and change within the context of a busy general practice.

### Sample size estimates

Based on a minimum expected effect size of 0.3 (from Benner et al. [14]), an estimated intra-class correlation coefficient (ratio of the variance *between* practices to the total variance (*within + between*) practices) of 0.03, a false positive error rate of 0.05 and statistical power of 0.8, we estimated that we would need to achieve a sample of 10 patients from each practice in each trial arm for the majority of the general practices in Stoke on Trent (target 46 of 55 practices – some practices did not have compatible software) [21]. This would allow us to detect a reduction in mean population CVD risk equating to about 5% of expected baseline level. Our original sample size calculations were based on a cluster randomised controlled trial design but, with the launch of the NHS Health Check programme and its national roll-out led by general practice just after the start of our evaluation, it was not feasible on ethical or practical grounds to continue with this design. On the basis that it was still of value to monitor the potential effects of implementation of the NHS Health Check programme and to estimate the value of additional lifestyle support, we decided to proceed with a modified trial whereby patients were randomised to groups within practices, accepting the potential losses of attribution to treatment and to statistical power that this would entail.

### Statistical analysis

Details of our proposed approach to analysis have been published previously [17]. In summary, multi-level regression modelling was used to evaluate differences in our primary outcome, 10-year CVD risk, between groups and over time, accounting for clustering at the practice level. Since the 10-year CVD risk variable was positively skewed, the data were log transformed for inclusion in the analysis. Individual level variables included in the model were age and gender, to account for possible variations in response to treatment for these factors [22-24]. Practice level variables included were socio-economic status of the practice population [25], practice size and staggered recruitment group (SRG). The latter was included to allow us to model the possibility of differences arising from experience with delivering the NHS Health Check service over time. Recruitment of practices and patients was staggered over effectively one year, which also included start-up and embedding of the local NHS Health Check service. The SRG variable allowed us to explore whether service effectiveness varied over time. A three level model was developed using the HML Software Version 6.08 (Scientific Software International, Lincolnwood, IL, USA). Level 1 variables were the repeated measurements within individuals by group and time (baseline and 1-year), individual (level 2) variables were age in years and gender and practice (level 3) variables were as described above. Analysis was performed on an intention-to-treat (ITT) basis, with last available measure carried forward where data were missing at follow-up.

Cardiovascular risk (log transformed) was modelled using a multi-level linear regression equation including variables with the following coding:

#### Practice level factors

• Socio-economic status, IMDG – 1 More deprived, 2 More affluent

• Staggered recruitment group, SRG – 1 Recruited in first group of practices… 5 Recruited in last group of practices

• Practice size, PRACSIZE – 1 Small (<3500 patients), 2 Medium (≥3500 and < 7000), 3 Large (≥7000)

#### Individual level factors

• Age (years), AGE

• Gender, SEX – 0 Male, 1 Female

#### Identifier variables related to

• Group, GROUP – 0 NHS Health Check only, 1 NHS Health Check plus additional lifestyle support

• Time, TIME – 0 Baseline, 1 Follow-up at 1 year.

The model also included variance components, r_0_ representing variation associated with differences between individuals within practices, u_00_ representing variation associated with differences between practices and e representing random (measurement) error.

The χ^2^ test was used to assess differences in categorical variables between groups. T-tests were used to test for differences between scale variables where the data were normally distributed, otherwise non-parametric tests were used. The Wilcoxon test and McNemar test (for binary outcomes) were used to assess changes in risk factor categories between baseline and the 1-year follow-up.

A p-value of < 0.05 was considered statistically significant in all tests and for regression model coefficients.

### Blinding

Researchers providing the intervention were masked to follow-up data and staff collecting data from patients were masked to treatment allocation. Participants received information as per usual practice from their general practitioner or practice nurse. The researcher responsible for statistical analysis (TC) was blinded to treatment allocation until all data entry and checking had been completed. Furthermore, all patient and practice identities were removed from any data included in the analysis.

## Results

**Figure 1:**
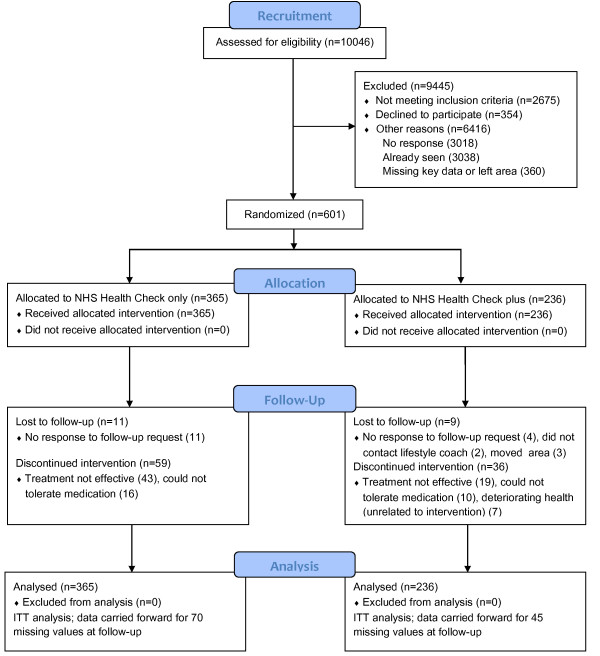
Summary of screening, recruitment, randomisation and flow of patients throughout the evaluation.

The flow of patients throughout the trial is summarised in Figure 1 and demographic details are displayed in Table 1. A much larger than anticipated number of patients contacted was ineligible for the trial (see Excluded box in Figure 1). The 6416 patients with other reasons for ineligibility included 3018 (30%) who did not respond to at least three invitations, 3038 (30.2%) patients who had already been seen by their GP and were receiving some form of treatment and 360 (3.6%) who were missing relevant details or had left the area. Eligible participants (n=601) were randomised into two groups, the NHS Health Check only group (n=365) and the NHS Health Check plus additional lifestyle support group (n=236). Baseline data were obtained for all participants, apart from 15 waist circumference and 6 pulse rate measurements. At follow-up, 70 (19%) participants from the NHS Health Check only group and 45 (19%) participants from the NHS Health Check plus additional lifestyle support group had missing CVD risk scores (primary outcome). Since the primary analysis was to be carried out on an ITT basis, missing values were replaced by carrying forward the last available measurement. There were no significant differences between the two groups on any of the demographic measures, Table 1.

**Table 1:** Demographic characteristics of sample groups

**Characteristic**	**Health Check group**	**Health Check plus group**
Age (years) Mean (SD)	63.9 (6.5)	63.3 (6.4)^a^
Gender F	36 (9.9%)	32 (13.6%)^a^
M	329 (90.1%)	204 (86.4%)
Ethnicity White	355 (97%)	226 (95.8%)^a^
Other	10 (3%)	10 (4.2%)
Socio-economic status^b^ Deprived	161 (45.9%)	121 (52.2%)^a^
Intermediate	121 (34.5%)	68 (29.3%)
More affluent	69 (19.7%)	43 (18.5%)

^a^ No significant difference between groups; SD–standard deviation; F-Female, M-Male.

^b^ Deprived-IMD deciles 1–3; Intermediate-IMD deciles 4–7; More affluent-IMD deciles 8–10 [25].

Baseline and 1-year follow-up measures for both groups are compared in Table 2 on the basis of available data only (best estimates of ‘on treatment’ effect). There were no significant differences between the groups at baseline except for weight, BMI and pulse rate (NHS Health Check plus additional lifestyle support group were on average 2.4 kg heavier, ~1 kgm^-2^ greater and 4/minute higher respectively). This was confirmed in separate comparisons of change in CVD risk and individual risk factors between groups. In terms of the comparison of available follow-up data with baseline data, both groups showed similar beneficial reductions in risk factors: about 7 mmHg in systolic blood pressure, 4mmHg in diastolic blood pressure, 0.65 mmol/l in total cholesterol level, 0.5 in total cholesterol/ HDL ratio and 2 cm in waist circumference. Changes in HDL, weight and BMI were negligible, though a small significant reduction in BMI (overall) of 0.3 kgm^-2^ was noted. Changes in absolute CVD risk were similar in both groups, showing about a 10% reduction from mean baseline score, corresponding to relative risks of 0.89 (0.87 – 0.92) in the Health Check only group and 0.91 (0.88 – 0.94) in the Health Check plus additional lifestyle support group (comparing scores at 1 year with baseline).

**Table 2:** **Comparison of baseline and 1-year follow-up measures by group**^**a**^

	**Baseline**						**Follow-up**					
	Health Check group			Health Check plus group			Health Check group			Health Check plus group		
	Mean	SD	N	Mean	SD	N	Mean	SD	N	Mean	SD	N
CVD risk baseline (%)	32.9	9.7	365	31.9	10.0	236	29.4^d^	9.7	295	29.2^d^	10.1	191
Systolic blood pressure (mmHg)	146.0	17.0	365	144.4	16.2	236	138.3^d^	14.7	314	138.7^d^	14.6	204
Diastolic blood pressure (mmHg)	84.9	9.5	365	85.3	9.6	236	80.5^d^	8.8	314	81.5^d^	8.9	204
Total cholesterol (mmol/l)	5.7	0.9	365	5.7	0.9	236	5.0^d^	1.0	308	5.1^d^	1.0	197
HDL cholesterol level (mmol/l)	1.2	0.3	365	1.2	0.3	236	1.2	0.3	298	1.2	0.3	192
Total cholesterol/ HDL cholesterol ratio	4.8	1.0	365	4.9	1.1	236	4.2^c^	1.1	300	4.4^d^	1.1	192
Weight (kg)	82.6^b^	13.8	365	85.0	14.5	236	82.8	13.5	313	84.3	14.5	200
Body mass index (kgm^-2^)	27.5^b^	4.1	365	28.7	5.0	236	27.6	4.1	313	28.4^e^	4.9	200
Waist circumference (cm)	99.5	11.8	355	101.3	11.2	231	97.9^d^	10.7	280	99.1^d^	11.4	168
Pulse rate (/minute)	70.9^c^	11.4	362	74.8	12.0	233						
Fasting plasma glucose (mmol/l)	5.6	2.0	365	5.4	1.0	236						
Height (cm)	173.0	8.1	365	172.5	8.3	236						

^a^ Comparisons carried out on the basis of available data only.

^b^ Baseline comparison between groups, p < 0.05.

^c^ Baseline comparison between groups, p < 0.001.

^d^ Paired comparison (baseline versus 1-year follow-up) within group, p < 0.001.

^e^ Paired comparison (baseline versus 1-year follow-up) within group, p < 0.01.

A summary of the final fitted model is included in Table 3. At the practice level, only socio-economic status made a marginally significant contribution to explaining the variation in CVD risk, with more affluent practices having a tendency towards lower risk scores. As expected, older age and male gender were associated with increased CVD risk. There was no significant difference in CVD risk between comparator groups, whereas CVD risk at the 1-year follow-up was significantly lower than at baseline. On this basis, overall population mean CVD risk was reduced from 31.1% to 27.8%, a reduction of 10.5% of the baseline score, after adjusting for all other factors in the model.

**Table 3:** Summary of multi-level model fit to log transformed CVD risk data

	**Factor**	**Coefficient**	**Standard error**	**T-ratio**	**df**	**p-value**
	Intercept	2.512	0.154	16.322	34	<0.001
Practice factors	IMDG	−0.079	0.039	−2.021	34	0.051
	SRG	0.000	0.009	0.028	34	0.978
	PRACSIZE	−0.026	0.017	−1.546	34	0.131
Individual factors	AGE	0.017	0.002	9.091	598	<0.001
	SEX	−0.256	0.033	−7.658	598	<0.001
Grouping factors	GROUP	−0.007	0.023	−0.287	1194	0.774
	TIME	−0.111	0.015	−7.501	1194	<0.001
						
	**Variance components**		**Standard deviation**		**df**	**p-value**
r_0_	0.055		0.235		561	<0.001
e	0.036		0.189			
u_00_	0.002		0.042		34	0.027

CVD-cardiovascular disease; df-degrees of freedom.

Grouping variables: IMDG-socio-economic status; SRG-staggered recruitment group; PRACSIZE-practice size; AGE-age group; SEX-gender; GROUP-treatment; TIME-baseline or follow-up.

Variance components: r_0_-between individuals; u_00_-between practices; e-random error.

The variance component between practices, u_00_, though significant, was small, indicating that the variation in mean CVD risk between practices was small. The variance component between individuals within practices, r_0_, was larger and also significant.

Changes in the clinical measures between baseline and the 1-year follow-up are shown in Table 4. Comparisons in this case have been carried out on an ITT basis (best estimates of whole population effect). There were no significant differences between the groups on any of the measures. However, both groups showed significant and clinically valuable changes in CVD risk, systolic and diastolic blood pressure, total cholesterol, total/HDL cholesterol ratio and waist circumference. Weight, BMI and HDL were effectively unchanged.

**Table 4:** **Summary of changes (baseline – follow-up) in clinical measures (mean, 95% CI)**^**a**^

**Measure**	**NHS HC only**	**NHS HC plus**	**Overall**
CVD risk (%)	3.10^b^	2.80^b^	2.98^b^
	(2.43, 3.76)	(2.01, 3.58)	(2.47, 3.48)
Systolic blood pressure (mmHg)	6.65^b^	5.64^b^	6.25^b^
	(4.94, 8.36)	(3.74, 7.53)	(4.98, 7.53)
Diastolic blood pressure (mmHg)	3.56^b^	3.31^b^	3.47^b^
	(2.61, 4.52)	(2.25, 4.38)	(2.76, 4.18)
Total cholesterol (mmol/l)	0.54^b^	0.56^b^	0.55^b^
	(0.45, 0.64)	(0.44, 0.68)	(0.47, 0.62)
High density lipoproteins (mmol/l)	−0.01	−0.01	−0.01
	(−0.03, 0.00)	(−0.03, 0.01)	(−0.03, 0.00)
Total cholesterol/HDL ratio	0.42^b^	0.47^b^	0.44^b^
	(0.33, 0.51)	(0.35, 0.59)	(0.37, 0.51)
Weight (kg)	0.23	0.51	0.34
	(−0.24, 0.70)	(−0.21, 1.24)	(−0.06, 0.74)
Body mass index (kgm^-2^)	0.02	0.22^d^	0.10^c^
	(−0.13, 0.17)	(−0.03, 0.47)	(−0.04, 0.23)
Waist circumference (cm)	1.19^b^	1.61^b^	1.36^b^
	(0.60, 1.78)	(0.90, 2.32)	(0.90, 1.81)

CI-confidence interval; HC-Health Check; ^a^ Comparisons carried out on ITT basis; ^b^ p< 0.001; ^c^ p< 0.05; ^d^ p< 0.01.

Table 5 summarises the changes in relevant risk factors between baseline and follow-up. At the 1-year follow-up, there was significant reduction in the number of patients who had high blood pressure, high cholesterol and who were smokers. Obesity was not significantly changed but there was some evidence of beneficial population change, where central obesity was reduced in patients in the additional lifestyle support group and overall. There was further evidence of beneficial lifestyle change through improved diet and physical activity scores in both treatment groups (Wilcoxon test on ordered categories, p < 0.001). Mean diet score increased from 2.1 to 2.4 (NHS Health Check only group) and from 2.2 to 2.45 (NHS Health Check plus additional lifestyle support); mean physical activity score increased from 2.65 to 2.8 (NHS Health Check only group) and from 2.67 to 2.81 (NHS Health Check plus additional lifestyle support).

**Table 5:** Comparison of changes in risk factor classification by group

**Risk factor**	**Risk factor at baseline**	**NHS HC only**		**NHS HC plus**		**Overall**	
		**Risk factor at follow-up**					
		**No**	**Yes**	**No**	**Yes**	**No**	**Yes**
High blood pressure	No	251	20^a^	158	14^a^	409	34^a^
	Yes	61	33	35	29	96	62
High cholesterol	No	149	18^a^	81	9^a^	230	27^a^
	Yes	62	136	56	90	118	226
Smoking	No	158	10^a^	111	3^a^	269	13^a^
	Yes	46	151	29	93	75	244
Obese	No	271	11	147	6	418	17
	Yes	10	73	9	74	19	147
Central obesity	No	189	15	96	5^a^	285	20^a^
	Yes	28	123	25	105	53	228

^a^ p < 0.01, McNemar test.

NHS HC – NHS Health Check only; NHS HC plus – NHS Health Check plus additional lifestyle support.

## Discussion

### Main findings

Both groups of patients considered to be at high risk (≥ 20%) of having a cardiovascular event within the next 10 years showed significant and clinically valuable reduction in CVD risk and in established CVD risk factor profiles over the 1-year intervention period. These beneficial changes were similar in the two groups. Thus, there was no added value of additional lifestyle support, at least, in terms of absolute cardiovascular risk and individual CVD risk factor reduction. In principle, these findings support the effectiveness of the NHS Health Check programme delivered through general practice. However, as shown in Figure 1 (Excluded box), overall uptake was low, indicating perhaps that population readiness to make change was not as good as it ought to have been, given that all patients screened and contacted had been identified from practice records as being at high risk of experiencing a CVD event.

A part of this lack of population readiness to make change may be attributed to practice or community readiness to support change. We should bear in mind that this research was carried out during the early stages of the national roll-out of new policy. For many practices, this was the first opportunity they had had to have access to a full electronic search of the practice database and a complete list of current ‘high CVD risk’ patients to manage proactively. Although additional funding was made available through a Locally Enhanced Service agreement, there will undoubtedly have been a step change in internal demand within each practice and in external demand in the supporting community. Alternative modes of recruitment of patients may offer some potential for increased efficiency and reduction of costs. Some of these, for example bespoke drop-in clinics, opportunistic health checks or partial health checks, have been piloted in the overall programme of research in Stoke on Trent, of which this study is a part [26,27]. Coupled with general practice readiness, it is also true that the type of client represented by a high CVD risk patient may be quite different from the usual users of community services. We did not have sufficient resources to explore these aspects of service delivery more fully within this research but they would appear to warrant more detailed investigation.

The apparent lack of effectiveness of the lifestyle support programme could be due to the sub-optimal referral to the lifestyle coach, sub-optimal delivery of the services referred to or poor compliance of patients with the support sessions offered. Our data on referral to the lifestyle coaches and the personal health plan goals set by participants (not reported here) indicated that referral to the lifestyle coach was good and valued by participants. Thus, future research aimed at improving the contribution of additional lifestyle support for the reduction of CVD risk should focus on the delivery and compliance with treatment issues of the support services referred on to.

In general, data collection, data quality, retrieval and download were satisfactory for the NHS Health Check process to work through general practice. That is not to say that there were not some issues that need addressing. However, the majority of patients who responded to the invitation to attend for a vascular risk health check had their risk score corroborated by their clinical re-assessments.

### What is already known on this topic

Previous researchers have demonstrated that reductions in estimated CVD risk can be achieved with more concerted action to address multiple risk factors through general practice [14,16] or lifestyle behaviours [15] but our study is the first, we believe, to attempt to systematically embed multi-factorial risk reduction in general practice across a whole city. It has been proposed that such whole -of-system action will be needed to address the growing challenge of chronic diseases, including heart disease, cancer, stroke, diabetes and respiratory diseases [7,28].

The importance and multi-factorial nature of CVD risk are well established as is the multiplier effect of risk factor combinations. On the other hand, public acceptance and health practitioner acceptance of the need for population responsibility and action for change are not widespread. Given that the majority of the population is registered with and attends their doctor at least once annually, general practice is well positioned to influence patient choice and referral to treatment options and other community services.

### What this study adds

Routine scanning of electronic medical records to provide lists of high risk (for CVD in this instance) patients provides opportunity for systematic management of chronic disease populations through GP, and preventive measures therefor. Overall, data quality was adequate though most practice lists contained ‘ghost’ data and missing or non-usable items, requiring varying degrees of screening and cleaning before use. Approximately 8% of those on the lists drawn from practice records could not be re-contacted for administrative reasons, where either key data were missing or records were not updated, for example, for patients who had moved away from the area or who had died. A further approximately 29% of patients did not respond to the invitation to attend a health check (with a minimum of two follow-up invitations). This represents an area of uncertainty in that we have no way of knowing whether these patients were simply not interested in a health check and chose not to respond or whether the invitations were never received. Thus, about two thirds of those patients on lists compiled from practice databases were accessible and, potentially, could benefit from the NHS Health Check service. The error or uncertainty in the remaining one third of patients may imply an additional administrative burden for practices to routinely assure data entry, to check data for completeness and to archive redundant data.

Data entry would also benefit from greater standardisation and more robust supporting software (with in-built data checking where feasible). Free text entry, for example, was very cumbersome to analyse for many of the fields, including primary risk fields e.g. smoking status, used in this research and consumed a significant amount of resource simply to recode already entered data. Notwithstanding the loss of efficiency, this process is error prone and, bearing in mind that a single missing datum could mean that a significant diagnosis or treatment could be missed, would be best avoided.

### Limitations

The national policy directive and launch of the NHS Health Check service overtook our original cluster randomised controlled trial design (which had been piloted and planned in the two years preceding the national programme launch). In effect, the NHS Health Check service became the standard treatment for the population of interest in our trial. Thus, instead of being a randomised controlled trial it became a randomised comparison of two treatments, both of which were relatively new developments. This meant that we did not have a control group against which to compare our two treatments. This limits our ability to attribute effects to treatments.

In addition, uptake was lower than anticipated, even taking into consideration that the NHS Health Check service was being implemented simultaneously across the whole of general practice in Stoke on Trent. Thus, recruitment to the trial was much less than target (35 versus 46 practices target; 601 versus 1840 patients target). Notwithstanding this point, significant reductions (~10% of mean baseline score) were detected in both groups. We attribute this positive outcome to a combination of greater effect size for both treatments and a lower loss to follow-up (~20% actual versus 50% estimated).

The choice of risk estimator, having a limited set of risk factors, should also be considered. The Framingham 10-year CVD risk calculator, albeit the recommended approach at the time our research was carried out, includes just three modifiable risk factors: blood pressure, cholesterol levels and smoking. It is possible that this measure may lack the sensitivity to detect more subtle effects of lifestyle changes such as increased physical activity, better diet and weight loss that may take a longer time to manifest than the one year of follow-up considered here. This may explain, in part, our inability to detect an effect of the additional lifestyle support after one year.

About 20% of data were missing at follow-up. Whilst ITT analysis mitigates this problem to some extent, the fact remains that this adds a degree of uncertainty to effect size (and confidence interval) estimates obtainable from the research. Sophisticated methods of imputation of missing data are available [29,30]. We did consider imputation but rejected the idea because we felt that its technical complexity and improved precision would not justify the effort involved, given that, due to the launch of a national policy initiative part way through the trial, we were unable to maintain a control group for the study.

Both treatment groups were recruited from within the same practices. Each patient was recruited individually and independently but we have no way of knowing whether there was communication between patients in different treatment groups that may have led to treatment contamination. Furthermore, it was not feasible, within available resources, to monitor every treatment, and adherence to treatment, of every patient. Thus, we were unable to differentiate independent effects of individual treatments.

Finally, there was an inadvertent imbalance in the number of participants in the two arms of the trial. This came about because a number of patients withdrew from the study after having given their written informed consent but before they had left the clinic after their final baseline health check visit. This was an unforeseen circumstance and presented us with an ethical dilemma – patient choice or research rigour. We took the pragmatic decision that we could not go against the patient’s choice and so these patients were treated as if they had declined to participate in the research. There were no apparent differences in demographic and key clinical measures between our two groups but we have no way of knowing whether other important biases may have been introduced by this means. Certainly, there does seem to be a bias in treatment preference since the choice to opt out in this way was higher for those allocated initially to the additional lifestyle support group. This observation adds weight to our earlier point about there being a need to consider population readiness for lifestyle change more fully.

## Conclusions

Introduction of the NHS Health Check service in Stoke on Trent led to significant reductions in estimated population cardiovascular disease risk and associated individual risk factors. There was no further reduction in risk measures from the additional lifestyle support package offered to patients. Uptake of the service was lower than anticipated and this may have implications for the overall effectiveness (and cost-effectiveness) of this national policy initiative. On the other hand, routine screening of electronic medical records is viable and offers potential for the proactive and systematic management of population cardiovascular risk.

## Abbreviation

BMI: Body mass index (weight (kg)/height (m)^2^); CI: Confidence interval; CVD: Cardiovascular disease; df: Degrees of freedom; GP: General practice/ general practitioner; HC: Health Check; HDL: High density lipoprotein (cholesterol); IMD: Index of multiple deprivation (2010), an area-based measure of socio-economic status; ITT: Intention-to-treat analysis; NHS: National health service in the united kingdom; SD: Standard deviation; SRG: Staggered recruitment group (variable used to test the possibility of differences arising from experience with delivering the NHS Health Check service over time).

## Competing interests

The authors report no competing interests.

## Authors’ contributions

TC, RD, ZI, RC, YM contributed to conception and design. RC, YM, TC, JK, CG contributed to recruitment, coordination, delivery and data collection. TC, CG, JK were responsible for the primary analysis and all authors contributed to interpretation of the findings. TC drafted the manuscript and all authors have critically reviewed and contributed to subsequent revisions and have read and agreed the final manuscript.

## Authors’ information

ZI is Acting Director of Public Health, NHS Stoke on Trent. RC acted as Clinical Champion for the project. TC, RD were based at Staffordshire University at the start of the research.

## Funding

This work was supported by NHS Stoke on Trent as part of the local implementation of the NHS Health Check programme. The sponsor had input to the design of the study in that the evaluation was to be embedded in primary care and was to take at least the medium term (one year) view. Collection, analysis and interpretation of data, writing of the report and the decision to submit the paper for publication were entirely the responsibility of the authors.

## Pre-publication history

The pre-publication history for this paper can be accessed here:

http://www.biomedcentral.com/1471-2458/12/944/prepub
